# Mandibular advancement reduces pharyngeal collapsibility by enlarging the airway rather than affecting velopharyngeal compliance

**DOI:** 10.14814/phy2.15558

**Published:** 2023-02-09

**Authors:** Guilherme J. M. Garcia, Josiah J. Wolf, David A. Campbell, Ryan S. Bailey, Rodney A. Sparapani, Charles M. Welzig, B. Tucker Woodson

**Affiliations:** ^1^ Department of Otolaryngology and Communication Sciences Medical College of Wisconsin Milwaukee Wisconsin USA; ^2^ Joint Department of Biomedical Engineering Marquette University & The Medical College of Wisconsin Milwaukee Wisconsin USA; ^3^ Division of Biostatistics Medical College of Wisconsin Milwaukee Wisconsin USA; ^4^ Department of Medicine Tufts University School of Medicine Boston Massachusetts USA

**Keywords:** closing pressure, drug‐induced sedated endoscopy, mandibular advancement device, obstructive sleep apnea, oral appliance, pharyngeal compliance, tube law, upper airway collapsibility

## Abstract

Mandibular advancement devices (MADs) are frequently prescribed for obstructive sleep apnea (OSA) patients, but approximately one third of patients experience no therapeutic benefit. Understanding the mechanisms by which MADs prevent pharyngeal collapse may help optimize MAD therapy. This study quantified the relative contributions of changes in airspace cross‐sectional area (CSA) versus changes in velopharyngeal compliance in determining MAD efficacy. Sixteen patients with moderate to severe OSA (mean apnea–hypopnea index of 32 ± 15 events/h) underwent measurements of the velopharyngeal closing pressure (*P*_CLOSE_) during drug induced sedated endoscopy (DISE) via stepwise reductions in nasal mask pressure and recording of the intraluminal pressure with a catheter. Airspace CSA was estimated from video endoscopy. Pharyngeal compliance was defined as the slope of the area–pressure relationship of the velopharyngeal airspace. MAD therapy reduced *P*_CLOSE_ from a median of 0.5 cmH_2_O pre‐advancement to a median of −2.6 cmH_2_O post‐advancement (*p* = 0.0009), increased the minimal CSA at the velopharynx by approximately 20 mm^2^ (*p* = 0.0067), but did not have a statistically significant effect on velopharyngeal compliance (*p* = 0.23). *P*_CLOSE_ had a strong correlation with CSA but did not correlate with velopharyngeal compliance. Our results suggest that MADs reduce velopharyngeal collapsibility by increasing airway size as opposed to affecting velopharyngeal compliance. This contradicts the speculation of previous literature that the effectiveness of MADs is partially due to a reduction in velopharyngeal compliance resulting from stretching of the soft palate. These findings suggest that quantification of velopharyngeal CSA pre‐ and post‐MAD advancement has potential as a biomarker to predict the success of MAD therapy.

## Graphical abstract

This study quantified the relative contributions of changes in airway size versus changes in velopharyngeal compliance in determining the efficacy of mandibular advancement devices (MADs). Our results suggest that MADs reduce upper airway collapsibility by increasing airway size as opposed to affecting velopharyngeal compliance. These findings suggest that quantification of airway size pre‐ and post‐MAD advancement has potential as a biomarker to predict the success of MAD therapy.

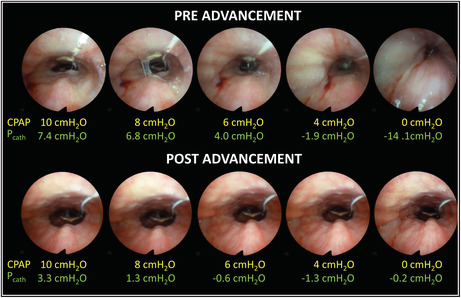

## 1 INTRODUCTION

Mandibular advancement devices (MADs), also known as oral appliances, are frequently prescribed for obstructive sleep apnea (OSA) patients who failed continuous positive airway pressure (CPAP) treatment. Despite being the leading treatment alternative to CPAP, MADs have a negligible therapeutic effect in approximately one third of patients (Sutherland et al., 2014, 2015; Van et al., 2022). To date, there is no widely used objective method to prospectively predict which OSA patients will benefit from MAD treatment. Therefore, understanding the impact of MADs on upper airway (UA) collapsibility is critical to achieve greater precision in personalized medicine.

The goal of MAD treatment is to enlarge the pharynx airspace by displacing the mandible forward. Several studies have demonstrated that MADs improve pharyngeal patency by quantifying their effect on gold standard measures of UA collapsibility, such as the pharyngeal critical pressure (*P*_CRIT_) and the pharyngeal closing pressure (*P*_CLOSE_) (Ayuse et al., 2006; Bamagoos et al., 2019; Edwards et al., 2016; Inazawa et al., 2005; Isono et al., 1995; Kato et al., 2000; Marques et al., 2019; Ng et al., 2003, 2006; Oliven et al., 2009). However, UA collapsibility is regulated by a complex interaction between airspace cross‐sectional areas (CSAs), neuromuscular factors, tissue stiffness, and the degree of negative intraluminal pressures generated by breathing. Due to the multifactorial nature of OSA, the precise mechanism by which MADs reduce UA collapsibility remains unclear. It is well documented that mandibular advancement increases the airspace CSA in the velopharynx (Ferguson et al., 1997; Hiyama et al., 2003; Isono et al., 1995; Isono, Tanaka, et al., 1997; Ryan et al., 1999). This increase in airspace CSA reduces airflow resistance thus lessening the magnitude of negative intraluminal pressures required for inhalation. However, the effect of MADs on pharyngeal compliance remains unclear. Several studies speculated that MADs increase tissue stiffness by stretching the soft palate, which would contribute to a reduction in UA collapsibility (Inazawa et al., 2005; Isono et al., 1995; Isono, Tanaka, et al., 1997; Kato et al., 2000). These studies hypothesized that this link between anterior tongue displacement and soft palate stiffness is mediated by the soft palate's connection to the base of the tongue via the palatoglossal arches (Isono, Tanaka, et al., 1997).

The impact of MADs on pharyngeal compliance has been quantified by only one study to date. In a group of 14 OSA patients, Oliven et al. (2009) reported that MADs had no effect on velopharyngeal compliance and interestingly increased oropharyngeal compliance (Oliven et al., 2009). More studies are needed to quantify the relative contributions of increases in airspace CSA and changes in pharyngeal compliance as it relates to the effectiveness of MAD treatment. The objective of this study is to quantify how MADs affect UA collapsibility by measuring the velopharyngeal compliance and closing pressure in adult OSA patients during drug induced sedated endoscopy (DISE).

## 2 MATERIALS AND METHODS

### 2.1 Patient selection

This project was approved by the Institutional Review Board of the Medical College of Wisconsin. OSA patients who failed CPAP treatment and were scheduled to undergo DISE for surgical planning were invited to participate in the research. The inclusion criteria were age above 18 years old, adequate dentition to support the MAD, no history of radiation therapy of the head & neck, no history of major ablative surgery of the UA, and no history of severe temporomandibular joint (TMJ) disorders. All patients completed verbal and written informed consent. Patients performed a polysomnography and completed the Epworth Sleepiness Scale (ESS) questionnaire, as well as the Nasal Obstruction Symptom Evaluation (NOSE) as part of their routine clinical workup (Johns, 1991; Stewart et al., 2004). The polysomnography provided the apnea–hypopnea index (AHI) quantified based on 1b criteria, the apnea index (AI), the hypopnea index (HI), and the fraction of respiratory events that were hypopneas (*F*_hypopnea_), which is defined as *F*_hypopnea_ = HI/AHI. A cohort of 16 patients participated in the study of whom 10 had an at‐home polysomnography and six had an in‐laboratory polysomnography.

### 2.2 In vivo measurements

DISE was performed in a semi‐dark, silent operating theater with the patient lying in supine position. The patient's head was kept at a neutral position with the Frankfort horizontal line perpendicular to the floor without neck rotation throughout the procedure. Propofol was used to induce sedation. Following application of Standard American Society of Anesthesia monitors for anesthetic administration, propofol was started and maintained at an infusion rate of 120–140 μg/kg/min via a peripheral intravenous line placed preoperatively. Supplemental propofol bolus doses of 10 mg intravenous approximately every minute were administered to achieve the target level of bispectral index (BIS) 65–70. The propofol dose was monitored and titrated by the anesthesiologist to maintain Ramsay 5 sedation (i.e., sluggish response to light glabellar tap or loud auditory stimulus). No other sedation medication was administered. However, all patients received 0.2 mg of glycopyrolate intravenous pre‐procedurally as an antisialagogue and 4% lidocaine via nasal pledgets in the operating room for topical anesthesia to facilitate placement of the endoscope.

**FIGURE 1:**
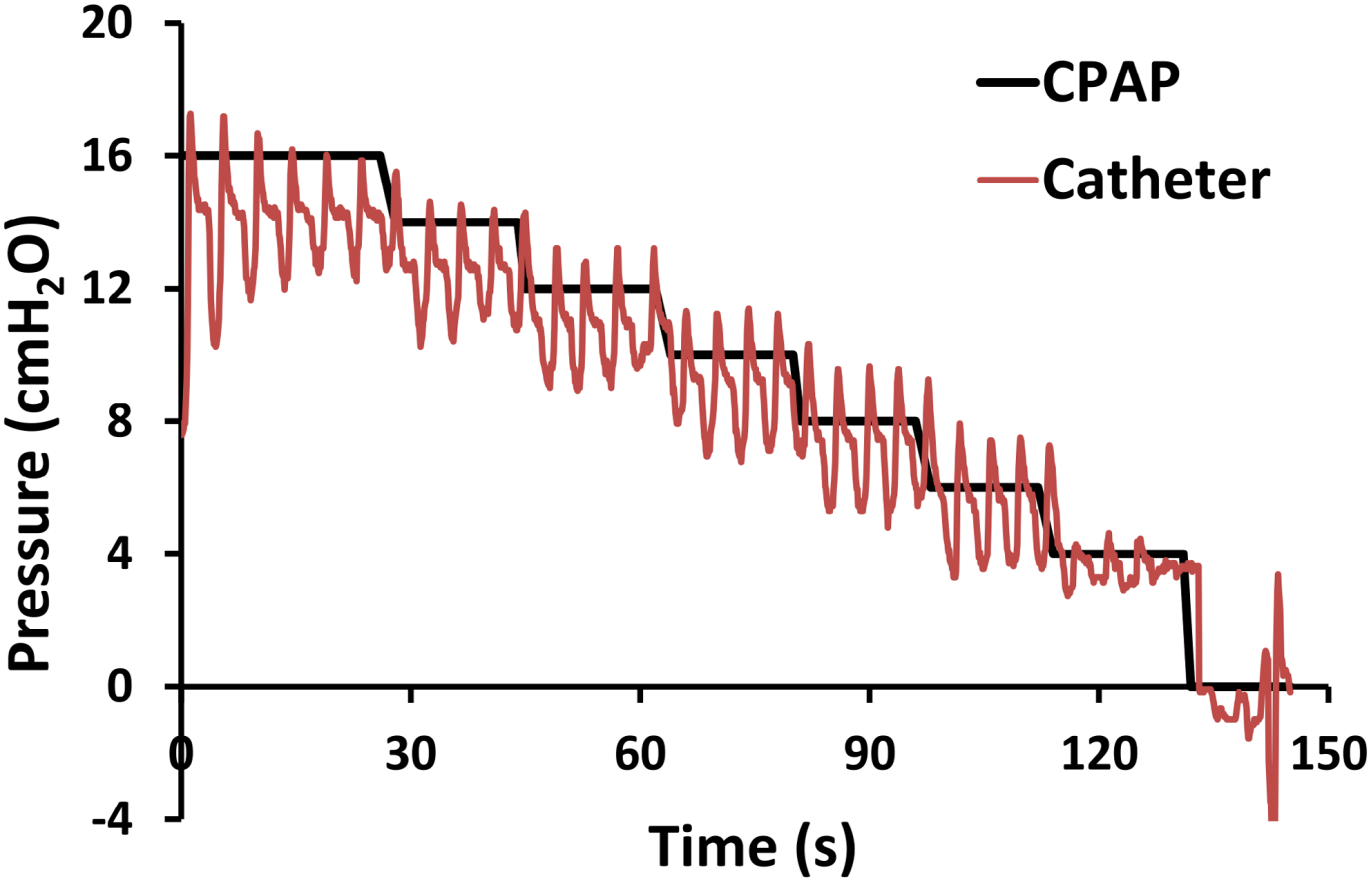
Protocol to measure the velopharyngeal closing pressure. The nasal mask pressure (CPAP pressure) was reduced in steps of 2 cmH_2_O starting from a holding pressure of 16 cmH_2_O until CPAP was turned off. Air pressure at the velopharynx was recorded with a pressure catheter.

**FIGURE 2:**
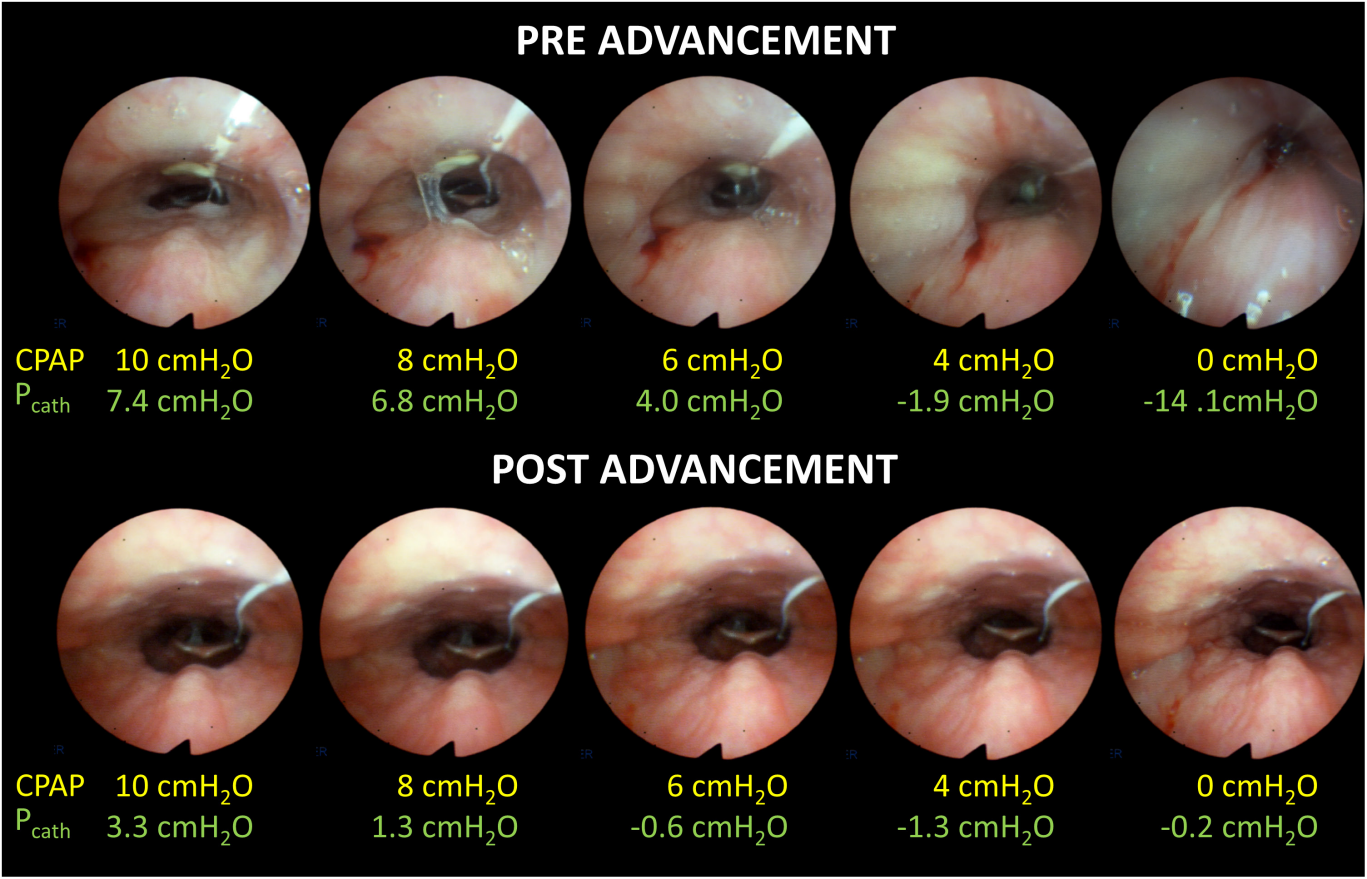
Endoscopic view of the velopharynx pre‐ and post‐ mandibular advancement at various nasal mask pressures in a representative OSA patient. The nasal mask pressure (CPAP) and mid‐inspiration catheter pressure (*P*_cath_) corresponding to each image are shown in yellow and green, respectively.

A disposable pressure catheter (Mikro‐Cath, Millar Inc.) was inserted through the nasal cavity. The tip of the pressure catheter, where the pressure sensor is located, was positioned in the velopharynx to measure intraluminal pressure at the site of collapse. A CPAP device was used to modulate air pressure at the nostrils via a nasal mask. Mouth closure was monitored by the surgeon performing the DISE. Air leaks through the mouth were identified by the surgeon and were addressed successfully with mouth closure and lip sealing so that no leak was observed during measurements. The active protocol was used to measure the velopharyngeal closing pressure (active *P*_CLOSE_). At the start of the experiment CPAP was raised to a holding pressure of about 16 cmH_2_O and then CPAP was progressively lowered in steps of 2 cmH_2_O until it was turned off (Figures 1 and 2). The minimal nasal mask pressure delivered by the CPAP was 4 cmH_2_O, which was then lowered to 0 cmH_2_O by turning off the CPAP. The pressure catheter, CPAP device, and endoscope video were connected to a data acquisition system (Alice 5, Phillips Respironics) to record the synchronized signals.

Mandibular advancement was performed with the myTAP Thornton Adjustable Positioner oral appliance (Airway Management, Inc.). This device allows incremental advances of the mandible by turning a knob. The maximum comfortable protrusion was identified when the patient was awake. The protocol was first completed with the MAD in the neutral position to measure *P*_CLOSE_ pre‐advancement. Then, the MAD was advanced to 75% of the maximum comfortable protrusion and the protocol was repeated to measure *P*_CLOSE_ post‐advancement.

**FIGURE 3:**
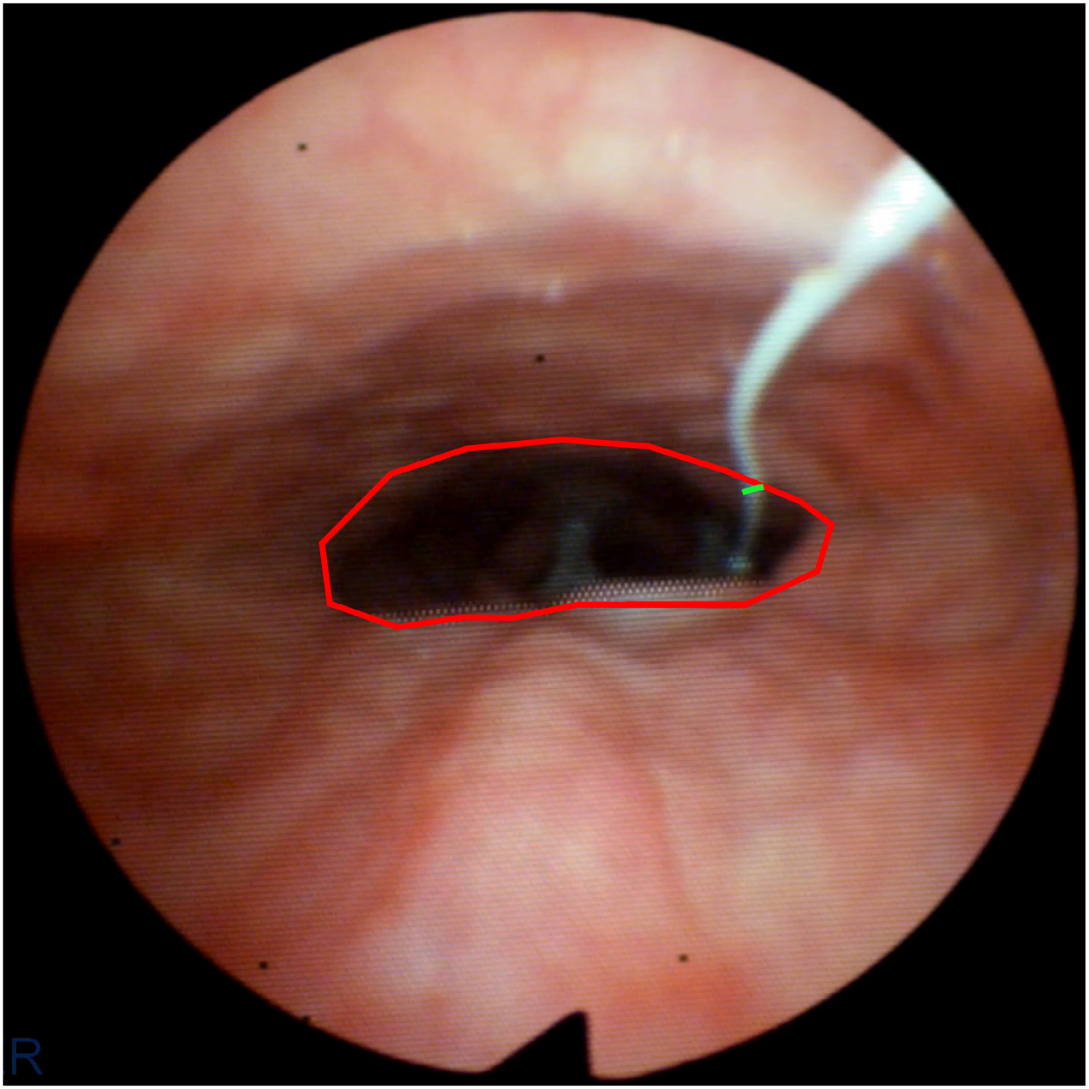
Estimation of the minimal airspace cross‐sectional area (CSA) at the velopharynx. The airway perimeter was outlined by hand (red color) and the number of pixels inside the perimeter was computed. Conversion from number of pixels to CSA in mm^2^ was based on the known diameter of the pressure catheter (green color).

The minimal airspace CSA of the velopharynx was estimated from the endoscope video. A code was developed in Matlab™ (MathWorks) which allowed the user to open the video, select a video frame, and manually outline the airway perimeter (Figure 3). The number of pixels inside the airway perimeter was counted. Conversion from number of pixels to CSA in mm^2^ was based on the shaft diameter of the Mikro‐Cath pressure catheter (0.77 mm) which was also measured in pixels in each video frame (Figure 3).

### 2.3 Calculation of the velopharyngeal *P*_CLOSE_

**FIGURE 4:**
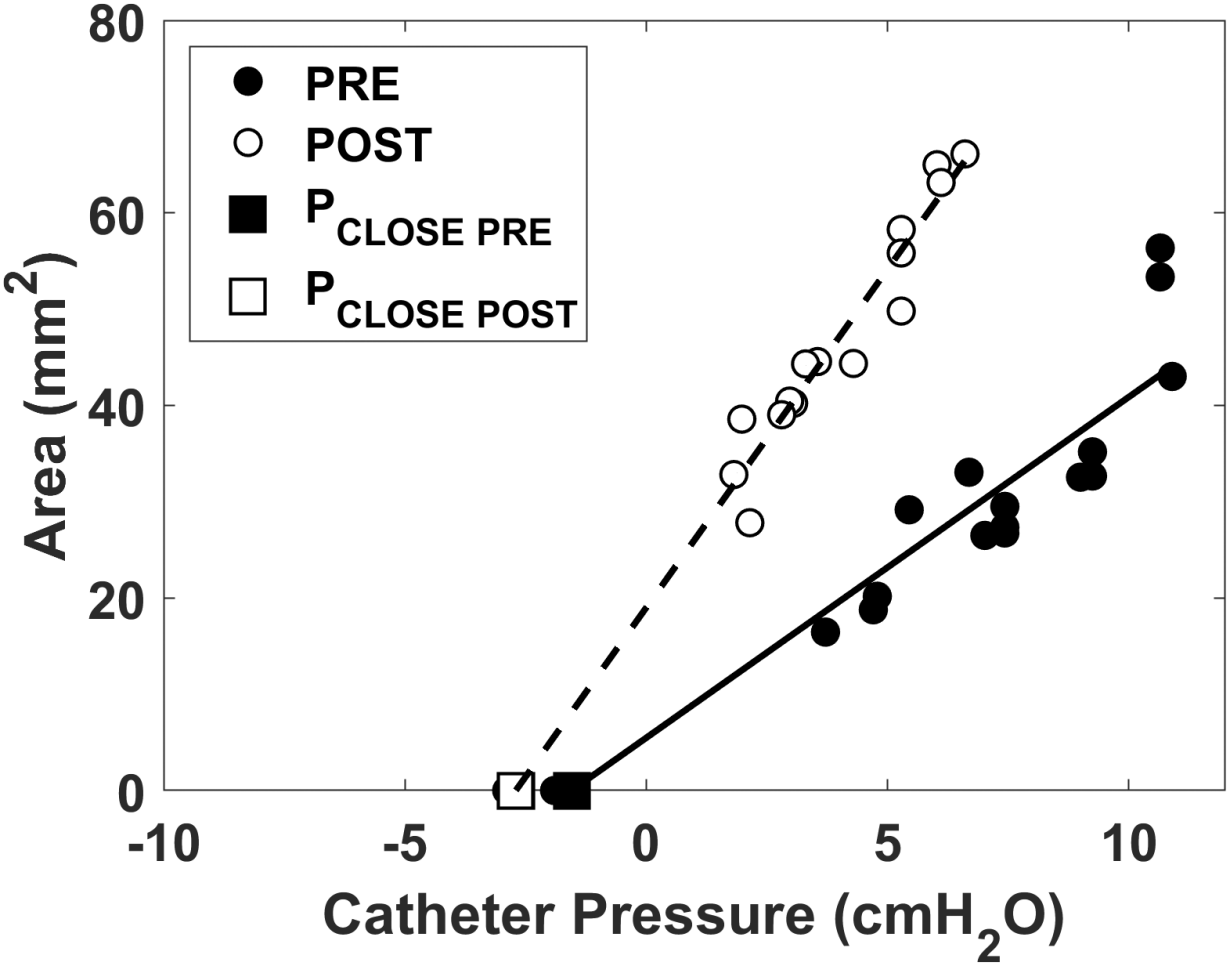
Area–pressure relationship of the velopharynx in a representative OSA patient (patient 3) pre‐ and post‐mandibular advancement. The pharyngeal closing pressure (*P*_CLOSE_) was estimated using a linear fit to estimate the intraluminal catheter pressure at which the velopharynx closes.

**FIGURE 5:**
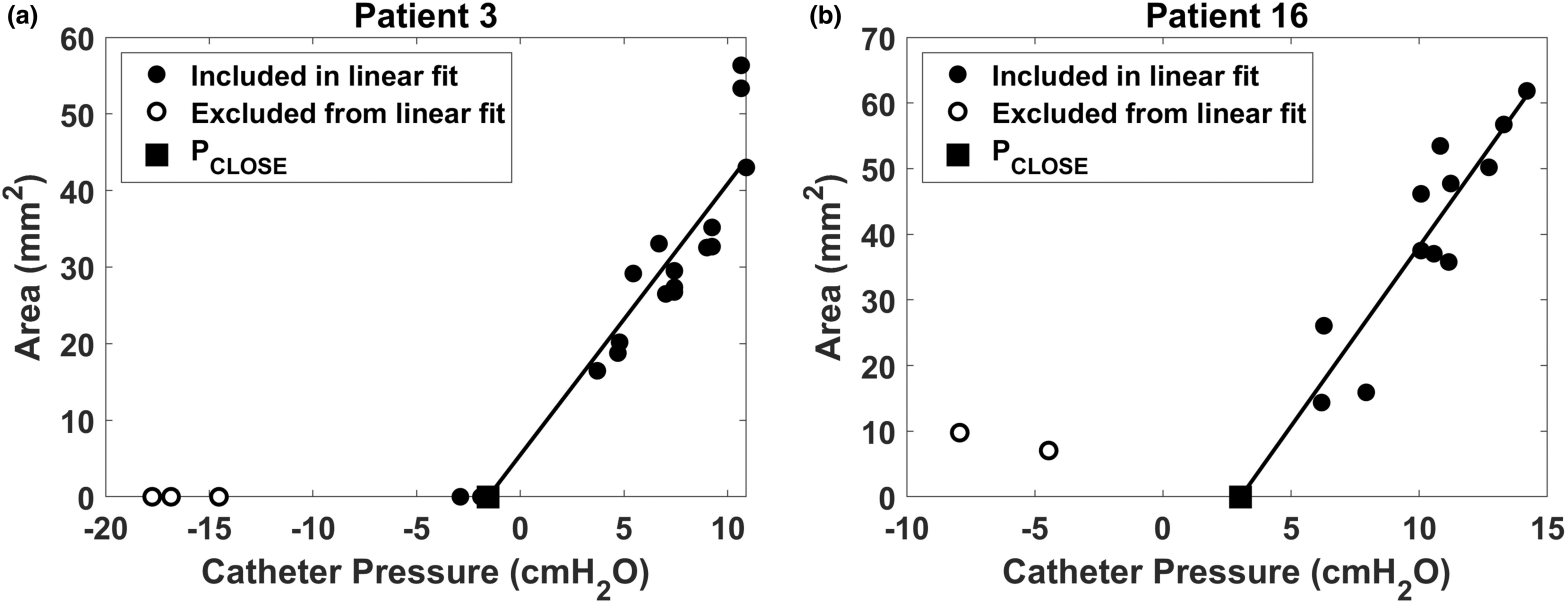
The pharyngeal closing pressure (*P*_CLOSE_) was estimated with a linear fit to the linear region of the area–pressure relationship. Data points clearly outside the linear region were excluded from the linear fit. (a) Patient 3 pre‐mandibular advancement. (b) Patient 16 pre‐mandibular advancement.

The area–pressure relationship was quantified at mid‐inspiration at the nadir of the catheter pressure during the breathing cycle. The area–pressure relationship was linear (Figure 4), namely

$$A=A_{P0}+C\times P_{\mathrm{VP}}\;\left(1\right)$$

where A is the minimal airspace CSA at the velopharynx, $P_{\mathrm{VP}}$ is the intraluminal pressure at the velopharynx measured with the pressure catheter, $A_{P0}$ is the intercept (the area of the velopharynx at zero gauge pressure, that is, when intraluminal pressure is equal to atmospheric pressure), and C is the velopharyngeal compliance (i.e., $C=\frac{\mathrm{dA}}{dP_{\mathrm{VP}}}$ is the slope of the area–pressure relationship). For each patient, a linear regression of the area and pressure measurements was performed to estimate the unknows $A_{P0}$ and C. In some cases (5 of 16 patients pre‐mandibular advancement and 3 of 16 patients post‐mandibular advancement), a few data points were observed outside the linear region of the area–pressure relationship (Figure 5). This occurred when the tip of the pressure catheter was located downstream of the choke point, so that very negative intraluminal pressures were recorded when the airway was closed (Figure 5a) or nearly closed (Figure 5b). Data points that were clearly outside the linear region were excluded from the linear regression (Figure 5).

The velopharyngeal closing pressure ($P_{\text{CLOSE}}$) is the local intraluminal pressure at which the area is zero (i.e., $P_{\mathrm{VP}}=P_{\text{CLOSE}}$ when A=0; see Figure 4). Substituting A=0 in Equation 1, we have

$$P_{\text{CLOSE}}=-\frac{A_{P0}}{C}\;\left(2\right)$$

**FIGURE 6:**
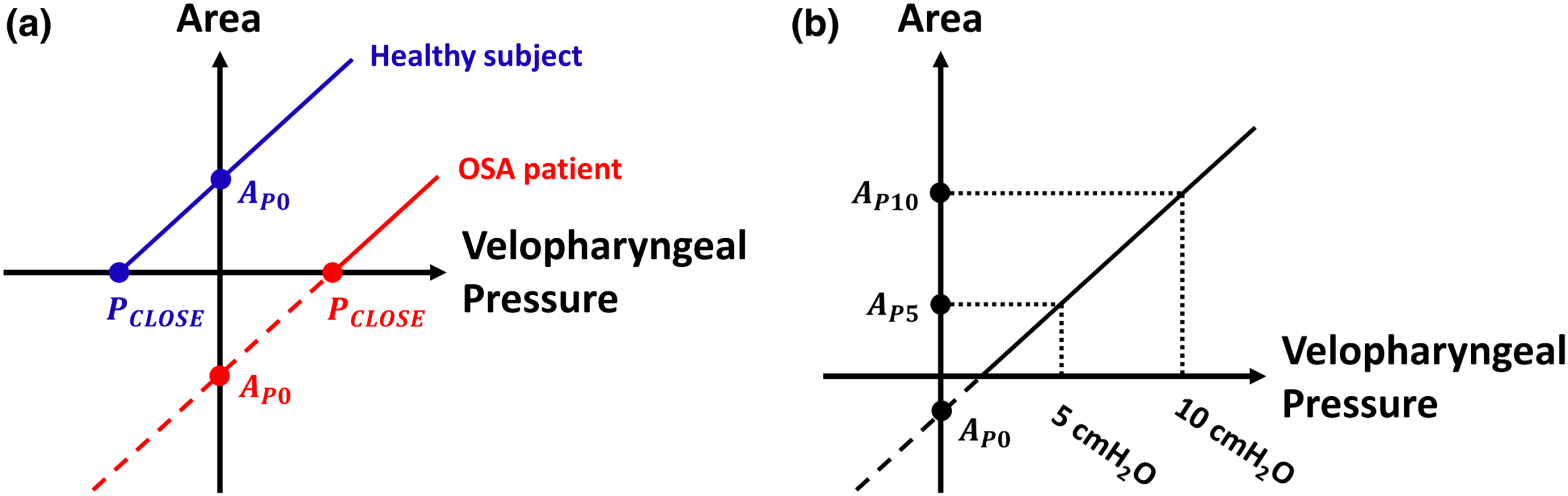
(a) Diagram illustrating a hypothetical healthy subject with negative *P*_CLOSE_ and positive intercept *A*_P0_ and a hypothetical OSA patient with positive *P*_CLOSE_ and negative intercept *A*_P0_. (b) Definition of the intercept *A*_P0_, the velopharyngeal area at intraluminal pressure of 5 cmH_2_O (*A*_P5_), and the velopharyngeal area at intraluminal pressure of 10 cmH_2_O (*A*_P10_).

This equation implies that the closing pressure is determined by the intercept, which represents airway size, and velopharyngeal compliance. The mechanical stability of the velopharynx can be improved by increasing the airway size or by reducing velopharyngeal compliance since both strategies reduce $P_{\text{CLOSE}}$. Note that $P_{\text{CLOSE}}$ and $A_{P0}$ have opposite signs because C is always positive. Thus, a negative closing pressure ($P_{\text{CLOSE}}<0$) corresponds to a positive $A_{P0}$ (Figure 6a). This is illustrated by a healthy subject whose velopharynx is open when the velopharyngeal pressure is equal to atmospheric pressure (i.e., A>0 when $P_{\mathrm{VP}}=0$). Conversely, a positive closing pressure ($P_{\text{CLOSE}}>0$) corresponds to a negative $A_{P0}$ (Figure 6a). This is illustrated by an OSA patient whose velopharynx is closed when the velopharyngeal pressure is equal to atmospheric pressure (i.e., A=0 when $P_{\mathrm{VP}}=0$). Naturally, the physical area cannot be a negative number. Therefore, the intercept $A_{P0}$ does not correspond to the physical area when $A_{P0}<0$. In patients with $A_{P0}<0$, the physical area ${\left(A_{P0}\right)}_{\text{physical}}$ is zero at $P_{\mathrm{VP}}=0$. Thus, the impact of MADs on the physical area of the velopharynx at zero intraluminal pressure ($P_{\mathrm{VP}}=0$) was computed by substituting ${\left(A_{P0}\right)}_{\text{physical}}=0$ when $A_{P0}<0$.

We also calculated the velopharyngeal area $A_{P5}$ corresponding to $P_{\mathrm{VP}}$ = 5 cmH_2_O and the area $A_{P10}$ corresponding to $P_{\mathrm{VP}}$ = 10 cmH_2_O (Figure 6b). From Equation 1, we have

$$A_{P5}=A_{P0}+C\times 5\;{\mathrm{cmH}}_{2}O\;\left(3\right)$$

$$A_{P10}=A_{P0}+C\times 10\;{\mathrm{cmH}}_{2}O\;\left(4\right)$$

The value of $A_{P5}$ was positive in all cases, except in one patient pre‐advancement of the MAD. The physical value of $A_{P5}$ was computed by substituting ${\left(A_{P5}\right)}_{\text{physical}}=0$ in the single case when $A_{P5}<0$. The value of $A_{P10}$ was positive in all patients before and after MAD advancement, thus ${\left(A_{P10}\right)}_{\text{physical}}=A_{P10}$.

### 2.4 Statistical analysis

The two‐sided Wilcoxon signed rank test was used to test the hypothesis that variables measured pre‐ and post‐mandibular advancement were statistically different at the level *p* < 0.05. The Wilcoxon rank sum test was used to test the hypothesis that variables were statistically different in hypopnea predominant versus apnea predominant patients at the level *p* < 0.05. The Pearson correlation coefficient was used to quantify the correlation between pairs of variables.

## 3 RESULTS

### 3.1 Patient population

A cohort of 16 OSA patients (2 females and 14 males) completed the research protocol. The patients had a median age of 58 years (interquartile range, IQR = [47, 61]), median AHI of 27 events/hour (IQR = [23, 40]), median AI of 7 events/hour (IQR = [4, 16]), median HI of 17 events/hour (IQR = [9, 22]), median *F*_hypopnea_ of 0.75 (IQR = [0.34, 0.83]), median body mass index (BMI) of 32 kg/m^2^ (IQR = [30, 34]), median ESS score of 12 (IQR = [8, 15]), and median NOSE score of 30 (IQR = [20, 53]) (Table 1).

**TABLE 1:** Demographic information of the study population.

Patient	Gender	Age (years)	AHI (events/hour)	AI (events/hour)	HI (events/hour)	*F*_hypopnea_	BMI (kg/m^2^)	ESS	NOSE
1	Female	51	6.5	0.3	6.2	0.95	35.2	12	30
2	Male	40	29.0	5.5	23.5	0.81	33.6	13	30
3	Male	44	27.4	15.6	11.8	0.43	36.4	17	50
4	Male	59	57.7	54.0	3.7	0.06	29.2	4	30
5	Male	65	25.5	8.9	16.6	0.65	30.6	11	5
6	Male	45	24.1	5.5	18.6	0.77	35.3	14	55
7	Male	59	34.4	9.6	24.8	0.72	30.4	20	70
8	Male	51	50.5	3.3	47.2	0.93	33.6	5	20
9	Male	64	24.3	4.0	20.3	0.84	31.5	16	25
10	Male	59	39.3	4.2	35.1	0.89	33.6	13	45
11	Female	60	20.0	15.4	4.6	0.23	24.8	2	15
12	Male	39	22.1	3.9	18.2	0.82	31.9	5	20
13	Male	61	26.2	13.5	12.7	0.48	29.8	17	35
14	Male	60	60.2	45.7	14.5	0.24	29.3	12	70
15	Male	53	22.0	4.2	17.8	0.81	32.9	10	5
16	Male	64	41.5	35.8	5.7	0.14	28.5	12	70
Average ± SD	—	54 ± 9	32 ± 15	14 ± 16	18 ± 11	0.61 ± 0.30	32 ± 3	11 ± 5	36 ± 22
Median	—	58	27	7	17	0.75	32	12	30
IQR	—	[47, 61]	[23, 40]	[4, 16]	[9, 22]	[0.34, 0.83]	[30, 34]	[8, 15]	[20, 53]

Abbreviations: AHI, apnea–hypopnea index, AI, apnea index, BMI, body mass index; *F*_hypopnea_, fraction of respiratory events that are hypopneas, ESS, Epworth Sleepiness Scale; HI, hypopnea index, NOSE, Nasal Obstruction Symptom Evaluation, SD, standard deviation, IQR, interquartile range.

### 3.2 Impact of mandibular advancement on velopharyngeal collapsibility

**FIGURE 7:**
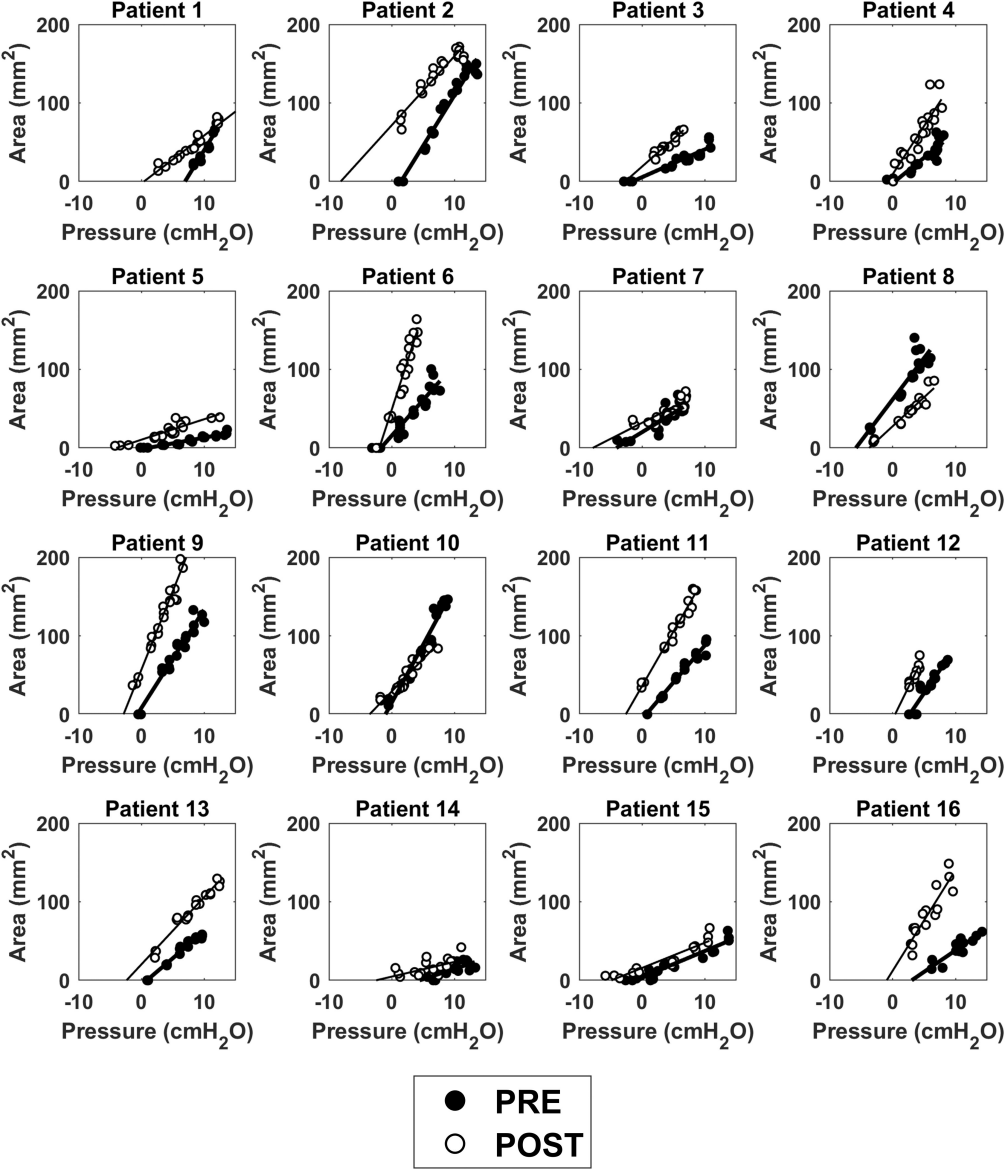
Area–pressure relationship at the velopharynx pre‐ and post‐mandibular advancement in 16 obstructive sleep apnea patients during drug induced sedated endoscopy.

**FIGURE 8:**
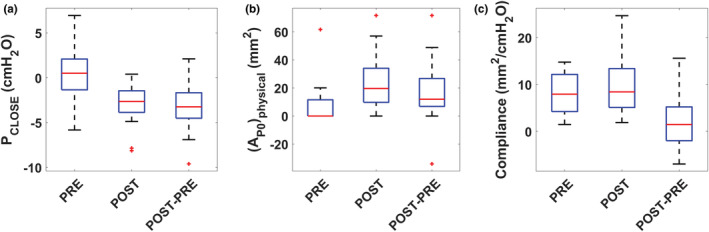
(a) Velopharyngeal closing pressure (*P*_CLOSE_), (b) velopharyngeal area at zero intraluminal pressure (${\left(A_{P0}\right)}_{\text{physical}}$), and (c) velopharyngeal compliance pre‐ and post‐mandibular advancement. The effect of mandibular advancement is shown as the difference between the post‐ and pre‐values.

Advancement of the MAD improved the mechanical stability of the UA by reducing *P*_CLOSE_ from a median of 0.5 cmH_2_O (IQR = [−1.4, 2.1]) pre‐advancement to a median of −2.6 cmH_2_O (IQR = [−3.9, −1.5]) post‐advancement (*p* = 0.0009) (Figure 7, Figure 8a, and Table 2). Advancement of the MAD enlarged the intercept $A_{P0}$ from a median of −1.9 mm^2^ (IQR = [−14.1, 11.6]) pre‐advancement to a median of 19.7 mm^2^ (IQR = [9.8, 34.1]) post‐advancement (*p* = 0.0038). The physical area at zero pressure increased from a median of 0 mm^2^ (IQR = [0, 11.6]) pre‐advancement to a median of 19.7 mm^2^ (IQR = [9.8, 34.1]) post‐advancement (*p* = 0.0067) (Figure 8b). Likewise, MAD advancement was associated with an increase in the velopharyngeal area at $P_{\mathrm{VP}}$ = 5 cmH_2_O and 10 cmH_2_O with ${\left(A_{P5}\right)}_{\text{physical}}$ increasing from a median of 29.2 mm^2^ (IQR = [15.7, 53.2]) pre‐advancement to a median of 67.5 mm^2^ (IQR = [42.1, 91.0]) post‐advancement (*p* = 0.0072) and ${\left(A_{P10}\right)}_{\text{physical}}$ increasing from a median of 65.9 mm^2^ (IQR = [39.1, 108.2]) pre‐advancement to a median of 108.9 mm^2^ (IQR = [65.7, 158.4]) post‐advancement (*p* = 0.011). In contrast, advancement of the MAD did not have a statistically significant effect on velopharyngeal compliance with a median compliance of 7.9 mm^2^/cmH_2_O (IQR = [4.2, 12.1]) pre‐advancement and 8.4 mm^2^/cmH_2_O (IQR = [5.1, 13.4]) post‐advancement (p = 0.23) (Figure 8c).

**TABLE 2:** Velopharyngeal closing pressure (*P*_CLOSE_), intercept (*A*_P0_), and velopharyngeal compliance (C) in 16 obstructive sleep apnea patients pre‐ and post‐mandibular advancement.

Patient	PRE			POST		
	*P*_CLOSE_ (cmH_2_O)	*A*_P0_ (mm^2^)	*C* (mm^2^/cmH_2_O)	*P*_CLOSE_ (cmH_2_O)	*A*_P0_ (mm^2^)	*C* (mm^2^/cmH_2_O)
1	6.9	−90.8	13.1	0.4	−2.4	6.1
2	1.5	−19.0	12.9	−8.1	71.7	8.8
3	−1.5	5.5	3.5	−2.7	19.0	7.0
4	0.3	−2.2	6.5	−0.7	9.1	12.3
5	1.1	−1.5	1.5	−4.0	10.5	2.6
6	−1.7	15.6	9.1	−2.0	48.8	24.7
7	−4.1	20.1	4.9	−7.9	32.0	4.1
8	−5.8	61.7	10.6	−3.7	27.5	7.4
9	−0.7	8.2	12.6	−2.9	57.0	20.0
10	−1.0	14.9	14.8	−3.5	29.2	8.3
11	0.7	−6.2	9.4	−2.6	36.1	13.9
12	2.7	−31.6	11.7	0.4	−6.4	16.3
13	0.9	−5.9	6.8	−2.4	20.3	8.6
14	4.4	−11.6	2.6	−2.5	4.7	1.9
15	−1.2	3.9	3.3	−4.9	15.7	3.2
16	3.0	−16.6	5.5	−0.9	12.0	12.9
Average ± SD	0.3 ± 3.1	−3.5 ± 31.3	8.0 ± 4.3	−3.0 ± 2.4	24.1 ± 21.4	9.9 ± 6.4
Median	0.5	−1.9	7.9	−2.6	19.7	8.4
IQR	[−1.4, 2.1]	[−14.1, 11.6]	[4.2, 12.1]	[−3.9, −1.5]	[9.8, 34.1]	[5.1, 13.4]

Abbreviations: IQR, interquartile range; SD, standard deviation.

**FIGURE 9:**
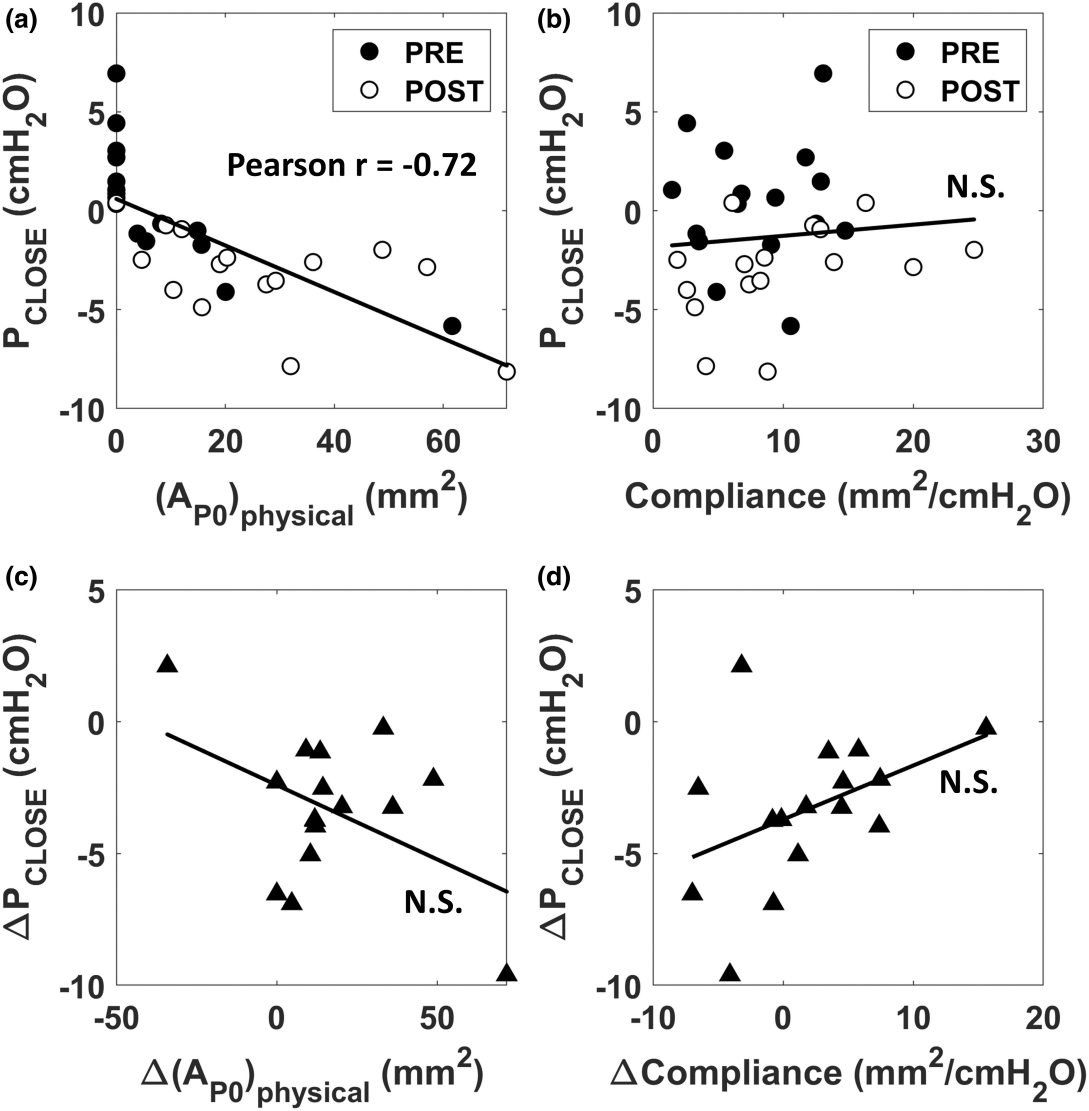
(a) Relationship between the velopharyngeal closing pressure (*P*_CLOSE_) and the velopharyngeal area at zero intraluminal pressure (${\left(A_{P0}\right)}_{\text{physical}}$). (b) Relationship between *P*_CLOSE_ and velopharyngeal compliance. (c) The reduction in *P*_CLOSE_ elicited by mandibular advancement did not correlate with the increase in airspace cross‐sectional area. (d) The reduction in *P*_CLOSE_ elicited by mandibular advancement did not correlate with changes in velopharyngeal compliance. Abbreviations: R = Pearson correlation coefficient, N.S. = not statistically significant, Δ = (post‐mandibular advancement)−(pre‐mandibular advancement).

The closing pressure had a strong correlation with the physical area of the velopharynx (Pearson *r* = −0.72, *p* < 0.0001) (Figure 9a). In contrast, the closing pressure did not correlate with the velopharyngeal compliance (Pearson *r* = 0.10, *p* = 0.60) (Figure 9b). The change in closing pressure after mandibular advancement, that is, Δ*P*_CLOSE_ = (*P*_CLOSE_)_PRE_ – (*P*_CLOSE_)_POST_, did not correlate with the change in the physical area of the velopharynx, that is, $∆{\left(A_{P0}\right)}_{\text{physical}}={\left(A_{P0}\right)}_{\text{physical}}^{\text{POST}}-{\left(A_{P0}\right)}_{\text{physical}}^{\mathrm{PRE}}$ (Pearson *r* = −0.47, *p* = 0.067) (Figure 9c). The change in closing pressure after mandibular advancement also did not correlate with the change in velopharyngeal compliance (Δ*C* = *C*_PRE_−*C*_POST_) (Pearson *r* = 0.43, *p* = 0.10) (Figure 9d).

### 3.3 Correlations between OSA severity and velopharyngeal collapsibility

**FIGURE 10:**
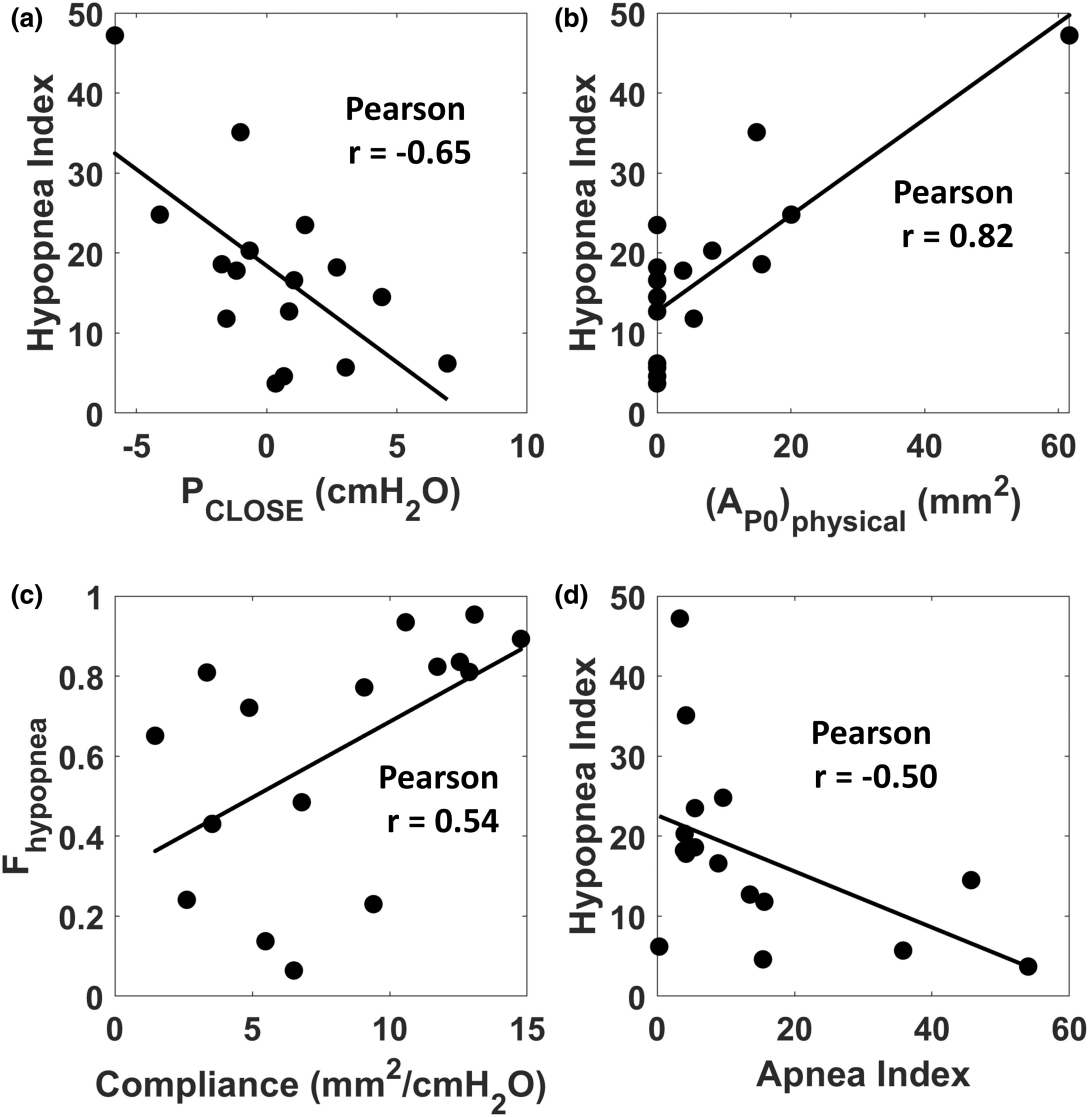
(a) Relationship between the hypopnea index and velopharyngeal closing pressure (*P*_CLOSE_). (b) Relationship between the hypopnea index and the velopharyngeal area at zero intraluminal pressure (${\left(A_{P0}\right)}_{\text{physical}}$). (c) Relationship between the fraction of respiratory events that were hypopneas (*F*_hypopnea_) and velopharyngeal compliance. (d) Relationship between the hypopnea index and the apnea index. *r* = Pearson correlation coefficient.

OSA severity as measured by the AHI had no correlation with metrics of velopharyngeal collapsibility (Table 3). However, OSA severity as measured by the HI correlated with the pre‐advancement velopharyngeal closing pressure (*r* = −0.65, *p* = 0.006) and the velopharyngeal area at zero intraluminal pressure (*r* = 0.82, *p* < 0.0001) (Figure 10a,b). In addition, velopharyngeal compliance correlated with the fraction of respiratory events that were hypopneas (*r* = 0.54, *p* = 0.033) (Figure 10C). Interestingly, the HI and AI were inversely correlated (*r* = −0.50, *p* = 0.05) so that patients with more apneas tended to have less hypopneas (Figures 10d). The AI correlated with AHI (*r* = 0.73, *p* = 0.001), *F*_hypopnea_ (*r* = −0.89, *p* < 0.0001), and BMI (*r* = −0.52, *p* = 0.04) (Table 4). The fraction of respiratory events that were hypopneas correlated with HI (*r* = 0.66, *p* = 0.005) and BMI (*r* = 0.67, *p* = 0.005). ESS score and NOSE score were correlated (*r* = 0.54, *p* = 0.03), which implies that patients with more severe subjective sleepiness also had more severe subjective nasal obstruction. Correlations between all other pairs of variables were not statistically significant (Tables 3 and 4).

**TABLE 3:** Pearson correlation coefficient (*r*) and *p* value between pairs of variables (*N* = 16 OSA patients).

	(*P*_CLOSE_)_PRE_		${\left(A_{0}\right)}_{\text{physical}}$		*C*_PRE_	
	*r*	*p*	*r*	*p*	*r*	*p*
AHI	−0.22	0.40	0.31	0.25	−0.27	0.32
AI	0.26	0.33	−0.31	0.25	−0.48	0.063
HI	**−0.65**	**0.006** *	**0.82**	**<0.0001** *	0.33	0.21
*F*_hypopnea_	−0.24	0.38	0.43	0.10	**0.54**	**0.033** *

*Note*: Statistically significant correlations are expressed in bold values.

Abbreviations: AHI = apnea–hypopnea index, AI = apnea index, HI = hypopnea index, *F*_hypopnea_ = HI/AHI = fraction of respiratory events that were hypopneas, *P*_CLOSE_ = velopharyngeal closing pressure, ${\left(A_{0}\right)}_{\text{physical}}$ = minimal airspace cross‐sectional area at the velopharynx, C = velopharyngeal compliance, PRE = pre mandibular advancement.

* Statistical significance at the level *p* < 0.05.

**TABLE 4:** Pearson correlation coefficient (*r*) and *p* value between pairs of variables (*N* = 16 OSA patients).

	AI		HI		*F*_hypopnea_		ESS		NOSE		BMI	
	*r*	*p*	*r*	*p*	*r*	*p*	*r*	*p*	*r*	*p*	*r*	*p*
AHI	**0.73**	**0.001** *	0.24	0.38	−0.47	0.06	−0.17	0.52	0.41	0.12	−0.27	0.32
AI	—	—	**−0.50**	**0.05** *	**−0.89**	**<0.0001** *	−0.21	0.43	0.41	0.11	**−0.52**	**0.04** *
HI			—	—	**0.66**	**0.005** *	0.08	0.77	−0.07	0.80	0.40	0.13
*F*_hypopnea_					—	—	0.22	0.42	−0.32	0.22	**0.67**	**0.005** *
ESS							—	–	**0.54**	**0.03** *	0.37	0.16
NOSE									—	—	0.01	0.97

*Note*: Statistically significant correlations are expressed in bold values.

Abbreviations: AHI, apnea–hypopnea index; AI, apnea index; BMI, body mass index; *F*_hypopnea_, fraction of respiratory events that were hypopneas; ESS, Epworth Sleepiness Scale; HI, hypopnea index; NOSE, Nasal Obstruction Symptom Evaluation.

* Statistical significance at the level *p* < 0.05.

**FIGURE 11:**
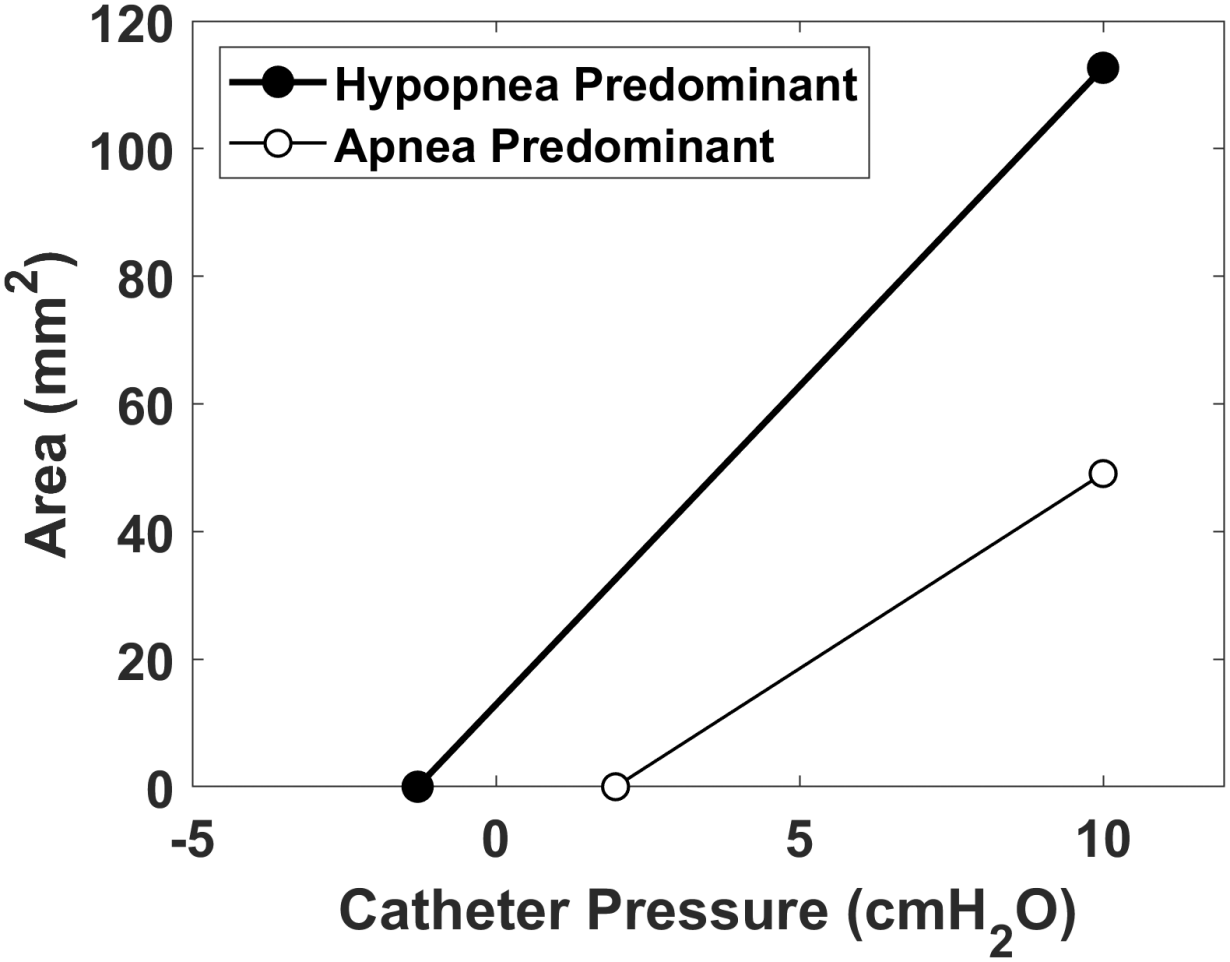
Tube law in hypopnea predominant and apnea predominant patients based on the average closing pressure and velopharyngeal compliance in each group (Table 5).

The inverse relationship between the apnea index and the hypopnea index (Figure 10d) suggested that OSA patients can be divided into two endotypes, namely patients with a predominance of apneas and patients with a predominance of hypopneas. To explore this possibility, we defined two groups based on the HI. Group 1 (hypopnea predominance) was composed of 8 patients with a HI above the cohort median (17.2 events/h). Group 2 (apnea predominance) was composed of eight patients with a HI below the cohort median. A comparison between the two groups revealed that patients with apnea predominance had a more positive *P*_CLOSE_ and a smaller ${\left(A_{0}\right)}_{\text{physical}}$, suggesting greater structural burden (Table 5). Figure 11 shows the tube law in the two groups based on the average closing pressure and average pharyngeal compliance in each group (Table 5). Figure 11 illustrates that the hypopnea predominant patients had a smaller *P*_CLOSE_ as predicted by the inverse relationship between *P*_CLOSE_ and the HI (Figure 10a).

**TABLE 5:** Polysomnography and upper airway collapsibility metrics in patients with a hypopnea index above the cohort median (group 1) and patients with a hypopnea index below the cohort median (group 2).

	Group 1: Hypopnea predominance (*n* = 8)	Group 2: Apnea predominance (*n* = 8)	*p*
AHI (events/h)	31 ± 10	33 ± 19	0.80
AI (events/h)	5 ± 2	24 ± 19	0.01*
HI (events/h)	26 ± 10	9 ± 5	0.0002*
*F*_hypopnea_	0.82 ± 0.07	0.40 ± 0.30	0.01*
(P_CLOSE_)_PRE_ (cmH_2_O)	−1.3 ± 2.7	2.0 ± 2.7	0.05*
${\left(A_{0}\right)}_{\text{physical}}$ (mm^2^)	15.5 ± 20.1	0.7 ± 1.9	0.01*
*C*_PRE_ (mm^2^/cmH_2_O)	10.0 ± 4.0	6.1 ± 3.8	0.13

Abbreviations: ${\left(A_{0}\right)}_{\text{physical}}$ = minimal airspace cross‐sectional area at the velopharynx, AHI, apnea–hypopnea index; AI, apnea index; C = velopharyngeal compliance; HI, hypopnea index, *F*_hypopnea_, fraction of respiratory events that were hypopneas, *P*_CLOSE_ = velopharyngeal closing pressure, PRE = pre‐mandibular advancement.

* Statistical significance at the level *p* < 0.05.

## 4 DISCUSSION

OSA is characterized by recurrent episodes of airflow limitation during sleep due to UA collapse. Interventions for OSA are aimed at improving the mechanical stability of the UA, which can be accomplished by enlarging the pharyngeal airspace or by reducing pharyngeal compliance. Several studies have reported that MADs improve the mechanical stability of the UA. MADs improve *P*_CLOSE_ by about 2 cmH_2_O during stage 2 natural sleep and by about 5 cmH_2_O during general anesthesia with total muscle paralysis according to our literature review (Table 6). Also, MADs improve *P*_CRIT_ by 4 to 6 cmH_2_O during natural sleep and by 5–9 cmH_2_O in sedated patients (Table 6). However, what remains unclear regarding how MADs improve pharyngeal collapsibility is the relative contribution of changes in airspace CSA and changes in pharyngeal compliance.

**TABLE 6:** Summary of previous studies that quantified the impact of mandibular advancement (MA) on upper airway collapsibility. The effect was statistically significant for all variables in all studies, except for the change in velopharyngeal compliance reported by Oliven et al. (2009).

Reference	Sample size	Degree of advancement	Physiological state	Metric	Before MA	After MA
Oliven et al. (2009)	14 OSA patients	MA performed manually to obtain maximum VP dilatation	Propofol‐anesthetized	C	16.5 ± 16.0 mm^2^/ cmH_2_O	14.5 ± 8.5 mm^2^/cmH_2_O
Isono et al. (1995)	13 OSA patients	MA performed manually by anesthetist	General anesthesia and total muscle paralysis	*P*_CLOSE_	1.5 ± 1.8 cmH_2_O	−8.1 ± 7.7 cmH_2_O
Kato et al. (2000)	37 SDB patients	MAD advanced 6 m	General anesthesia and total muscle paralysis	*P*_CLOSE_	2.2 cmH_2_O	−3.3 cmH_2_O
Ng et al. (2003)	10 OSA patients	MAD at maximum comfortable advancement	Stage 2 natural sleep	*P*_CLOSE_	−1.6 ± 0.4 cmH_2_O	−3.9 ± 0.6 cmH_2_O
Ng et al. (2006)	12 OSA patients	MAD at maximum comfortable advancement	Stage 2 natural sleep	*P*_CLOSE_	−1.1 ± 0.3 cmH_2_O	−2.8 ± 0.5 cmH_2_O
Inazawa et al. (2005)	Nine healthy subjects	MAD at 75% of maximum possible protrusion	Midazolam sedation	*P*_CRIT_	−4.2 ± 2.9 cmH_2_O	−13.3 ± 3.2 cmH_2_O
Ayuse et al. (2006)	Nine routine snorers—mild OSA patients	MAD at minimal position to prevent airflow limitations	Midazolam sedation	P_CRIT_	−1.9 ± 2.9 cmH_2_O	−7.3 ± 1.9 cmH_2_O
Oliven et al. (2009)	14 OSA patients	MA performed manually to obtain maximum VP dilatation	Propofol‐anesthetized	*P*_CRIT_	2.9 ± 2.2 cmH_2_O	−1.4 ± 2.9 cmH_2_O
Bamagoos et al. (2019)	12 OSA patients	MAD at maximum comfortable advancement	Natural sleep	*P*_CRIT_	1.8 ± 3.9 cmH_2_O	−4.0 ± 3.6 cmH_2_O
Marques et al. (2019)	25 OSA patients	MAD at maximum comfortable advancement	Natural sleep	*P*_CRIT_	−0.6 ± 1.9 cmH_2_O	−4.5 ± 2.7 cmH_2_O
Edwards et al. (2016)	14 OSA patients	MAD at maximal comfortable advancement	Non–REM natural sleep	*V*_passive_	1.9 ± 0.7 L/min	4.7 ± 0.6 L/min

Abbreviations: C, velopharyngeal compliance; MA, mandibular advancement; MAD, mandibular advancement device; OSA, obstructive sleep apnea; *P*_CLOSE_, velopharyngeal closing pressure; *P*_CRIT_, pharyngeal critical pressure; SDB, sleep disordered breathing; VP, velopharyngeal; *V*_passive_, ventilation at zero mask pressure estimated in passive P_CRIT_ protocol.

In our study, MADs improved *P*_CLOSE_ at the velopharynx by about 3 cmH_2_O during propofol sedation at Ramsay 5, which is consistent with the previous literature (Table 6). We also observed that advancing the MAD to 75% of comfortable protrusion increased the minimal airspace CSA at the velopharynx by approximately 20 mm^2^ (Table 2). A very similar result was observed by Ryan et al. (1999) with a CSA increase of 24 mm^2^ in the velopharynx when MADs were placed at maximum comfortable protrusion in mild to moderate OSA patients during natural sleep (Ryan et al., 1999). MAD therapy is known to produce a significant increase in UA volume (Marco‐Pitarch et al., 2021; Van et al., 2022; Marcussen et al., 2015) and a significant decrease in AHI (Edwards et al., 2016; Marques et al., 2019; Ng et al., 2006). Our study aligns with this previous literature and confirms that MADs improve airway patency and reduce pharyngeal collapsibility.

A novel contribution of our study is the observation that MADs have no effect on velopharyngeal compliance. Previous literature speculated that anterior displacement of the tongue would stiffen the soft palate through their connection via the palatoglossal arches, leading to a reduction in velopharyngeal compliance (Inazawa et al., 2005; Isono, Tanaka, et al., 1997; Isono et al., 1995; Kato et al., 2000). However, we found no statistically significant change on velopharyngeal compliance (Figure 8c). Substantial interindividual variability was observed with velopharyngeal compliance decreasing, remaining unchanged, or increasing after MAD advancement (Table 2). It is possible that interindividual differences in airway anatomy and physiology may explain the variable impact of MAD advancement on velopharyngeal compliance, but our data do not allow us to elucidate the source of this interindividual variability. Alterations in muscle activation after MAD advancement could theoretically change velopharyngeal compliance, but previous studies reported that MADs have no impact on muscle function (Bamagoos et al., 2019; Edwards et al., 2016). Interestingly, pharyngeal compliance is not affected by electrical stimulation of the genioglossus muscle (Oliven et al., 2007). Thus, our study suggests that in most patients the improvement in mechanical stability of the UA with MAD therapy is due to an increase in airway size, rather than a change in velopharyngeal compliance, in good agreement with Oliven et al. (2009).

When pharyngeal compliance is constant, *P*_CLOSE_ is linearly proportional to $A_{P0}$ (Equation 2) with an increase in the airspace CSA leading to a reduction in the closing pressure. *P*_CLOSE_ is closely related to the pharyngeal critical pressure (*P*_CRIT_), which is defined as the nasal mask pressure at which inspiratory airflow becomes zero. The two variables should have similar values in patients with a single site of collapse because the intraluminal pressure at which the airway closes (*P*_CLOSE_) should be similar to the nasal pressure at which airflow becomes zero (*P*_CRIT_) (Oliven et al. 2009). In the classical Starling resistor model, *P*_CRIT_ is interpreted as the external tissue pressure (Gold & Schwartz, 1996; Isono, 2012; Schwartz & Smith, 2013). Therefore, the reduction in *P*_CLOSE_ after MAD advancement can be interpreted as a reduction in tissue pressure at the site of collapse.

It is important to distinguish between compliance (i.e., the slope of the area–pressure relationship) of the pharyngeal airspace and the modulus of elasticity (i.e., the stress/strain ratio) of pharyngeal soft tissues. The modulus of elasticity is a material property that likely depends on muscle activation and its value measured in cadaver specimens is E≈ 1000 Pa (Birch & Srodon, 2009). Pharyngeal compliance depends on the modulus of elasticity (i.e., a soft material is more collapsible), but also depends on other factors, such as airway shape, wall thickness, and transmural pressure. A computational study of an UA model with a 2‐mm‐thick silicone pharynx reported that pharyngeal compliance had a complex power law relationship with the modulus of elasticity, namely $C\propto E^{-0.64}$ (Le et al., 2019). (Note that stiffer materials have higher E.) However, the modulus of elasticity of pharyngeal soft tissues in vivo remains poorly characterized (Subramaniam et al., 2016). Although we measured the pharyngeal compliance C, our methods do not allow us to quantify the modulus of elasticity E.

Our study is the first to describe the area–pressure relationship (tube law) of the pharynx in terms of the area at zero intraluminal pressure ($A_{P0}$). Previous studies focused on the maximal area (Isono et al., 1993; Isono, Remmers, et al., 1997) or the area where there is a change in the slope of the tube law (Oliven et al., 2010). Our results demonstrate that ${\left(A_{P0}\right)}_{\text{physical}}$ is zero in many OSA patients, which means that in these patients the UA during sleep is closed and that muscle activation via arousals is required to keep the airway open.

Our study is also the first to quantify the tube law at peak inspiration. Previous studies quantified the tube law at end‐expiration for two reasons. First, muscle forces are minimal at end‐expiration (Schwab et al., 1993), thus the end‐expiration tube law represents the passive behavior of the UA. Second, the flowrate is zero at end‐expiration, thus the entire UA is at the same intraluminal pressure. However, we were interested in quantifying the tube law at peak inspiration because UA collapse in OSA patients typically occurs at peak inspiration despite compensating muscle forces. In other words, we were interested in quantifying the “active tube law” in the presence of muscle forces as opposed to the “passive tube law” in the absence of muscle forces. A major challenge of quantifying the tube law at peak inspiration is that a steep pressure gradient arises in flow‐limited breaths, thus the magnitude of intraluminal pressure is dependent on the position of the pressure catheter in flow‐limited breaths. However, computational fluid dynamics (CFDs) and fluid–structure interaction (FSI) simulations show that a steep pressure gradient arises only after the airspace CSA drops below about 50% of its original value (Le et al., 2019; Lin et al., 2018). Thus, we expect that there was no flow limitation for CSAs above 50% of the maximum, so that the pressure gradient is small and the catheter position has a minor effect for those datapoints. Importantly, in most patients, we did not observe an obvious change in the slope of the tube law for CSAs below 50% of the maximum (Figure 7). As explained in Materials and Methods, we observed an S‐shaped tube law in a few patients, which we attributed to the pressure catheter being located downstream of the choke point (Figure 5b). In these cases, the linear region of the tube law was used to estimate the pharyngeal compliance. Consequently, the linear tube law reported in this study may not reflect the area–pressure relationship at the site of collapse in flow‐limited breaths.

Studies that investigated the tube law of the human pharynx in the absence of airflow using muscle paralysis or at end‐expiration reported that the tube law is exponential, that is, CSA plateaus at high pressures because the bony enclosure limits the expansion of soft tissues (Isono, Remmers, et al., 1997). For example, Isono et al. (1993) reported that the tube law of the velopharynx is well described by an exponential fit where the curve was approximately linear near *P*_CLOSE_, but approximately horizontal at high pressures (Isono et al., 1993). Oliven et al. (2010) fitted the tube law with two straight lines, namely a steeper line near *P*_CLOSE_ and a nearly horizontal line at higher pressures. We believe that we did not observe the plateau in our study because we did not investigate high enough pressures. In contrast, Genta et al. (2016) reported an S‐shaped tube law, which was similar to rubber tubes, where the initial high compliance region is followed by a higher stiffness region after contact of opposite walls (Kozlovsky et al., 2014; Zarandi et al., 2021). However, rather than describing the tube law in terms of the local pressure, Genta and coauthors measured epiglottic pressure. The relationship between velopharyngeal pressure and epiglottic pressure becomes highly nonlinear during flow limitation (Le et al., 2019), thus our interpretation is that the tube law is linear when the local pressure at the velopharynx is used, but it is S‐shaped when the downstream pressure at the epiglottis is used. This interpretation is consistent with the observation of an S‐shaped curve in our study in cases where the pressure catheter was downstream of the choke point (Figure 5).

An unexpected finding of our study was that P_CLOSE_ had no correlation with AHI but was inversely correlated with the HI (Table 3, Figure 10a). Previous studies have shown a moderate positive correlation between the AHI and the pharyngeal critical pressure (*P*_CRIT_), thus we expected a positive correlation between *P*_CLOSE_ and AHI. However, the only polysomnography metric that correlated with *P*_CLOSE_ was the hypopnea index (Table 3), which showed the opposite behavior, namely a higher *P*_CLOSE_ (more collapsible airway) was associated with fewer hypopneas (Figure 10a). To explain this unexpected finding, we noticed that the hypopnea index was inversely related to the apnea index (Figure 10d) so that more hypopneas were associated with fewer apneas. Edwards et al. (2014) reported that the fraction of respiratory events that are hypopneas (*F*_hypopnea_) is a key factor associated with high arousability. They measured arousability by measuring the nadir epiglottic pressure immediately preceding arousal. Patients with higher fraction of hypopneas had higher arousability (i.e., arousals occurred at lower levels of epiglottic pressure) (Edwards et al., 2014). In our study, patients with hypopnea predominance had significantly higher *F*_hypopnea_ as compared to patients with apnea predominance (0.82 ± 0.07 vs 0.40 ± 0.30, *p* = 0.01; Table 5), which suggests that patients with hypopnea predominance have higher arousability. We also observed a positive correlation between *F*_hypopnea_ and pharyngeal compliance (Figure 10c), which suggests higher arousability is correlated with higher pharyngeal compliance. We speculate that patients with higher pharyngeal compliance experience steeper (more sudden) pressure oscillations during flow‐limited breaths, which can stimulate pressure receptors in the airway and lead to an arousal. In contrast, patients with low arousability can activate UA dilator muscles to restore pharyngeal patency during sleep (Edwards et al., 2014). Thus, one expects that patients with low arousability have higher muscle tone, leading to lower pharyngeal compliance. In our study, patients with apnea predominance had lower pharyngeal compliance (suggesting higher muscle tone) but the difference was not statistically significant (Table 5). In summary, our finding that velopharyngeal compliance is correlated with *F*_hypopnea_ together with the correlation between *F*_hypopnea_ and arousability reported by Edwards et al. (2014) suggest a potential interplay between pharyngeal compliance and arousability. Additional studies are required to investigate this complex interaction between anatomical and physiological factors.

Our study has clinical implications regarding patient selection for MAD therapy. Our study suggests that the efficacy of MAD therapy is determined by the magnitude of change in velopharyngeal CSA rather than by a change in velopharyngeal compliance. Research studies have shown that the change in velopharyngeal CSA with MAD advancement can be quantified with endoscopy and that the MAD‐induced change in velopharyngeal CSA is predictive of MAD outcomes (Chan et al., 2010; Okuno et al., 2016; Ryan et al., 1999). Thus, our study supports the use of endoscopy‐based quantification of velopharyngeal CSA pre‐ and post‐MAD advancement as a biomarker to predict the success of MAD therapy.

Together with previous literature, our results also suggest that patients with lower *P*_CLOSE_ are more likely to respond to MAD therapy and provide a potential explanation for why MADs are unable to eliminate airflow limitation in about two‐thirds of patients (Sutherland et al., 2014, 2015; Van et al., 2022). It has been reported that *P*_CLOSE_ at the velopharynx is about −3.8 ± 3.4 cmH_2_O in healthy subjects, 0.9 ± 1.3 cmH_2_O in patients with mild OSA, and 2.8 ± 2.8 cmH_2_O in patients with severe OSA (Isono, Remmers, et al., 1997). Furthermore, the spectrum of P_CRIT_ values has been reported as being below −8 cmH_2_O in healthy subjects, between −8 and − 4 cmH_2_O in snorers, between −4 and 0 cmH_2_O in patients with obstructive hypopneas, and above 0 cmH_2_O in patients with obstructive apneas (Gold & Schwartz, 1996). Our literature review indicates that the effect size of MADs is to reduce *P*_CLOSE_ by about 2 cmH_2_O and to reduce *P*_CRIT_ by 4 to 6 cmH_2_O during natural sleep (Table 6). This suggests that the effect size of MADs is insufficient to prevent UA collapse in patients with high P_CLOSE_.

Several limitations of this study must be acknowledged. First, the small sample size (*n* = 16) may hinder generalization of our results. While the prevalence of OSA is higher in males, OSA has a high incidence in both men and women (Young et al., 1993). Our sample contained primarily men and does not provide insight on the phenotypic variations in UA collapse that may exist between males and females.

Second, pharyngeal CSAs were estimated by outlining the airway perimeter in endoscopic video frames (Figure 3). While this technique has been used in previous studies of UA biomechanics (Isono et al., 1993; Isono, Remmers, et al., 1997; Oliven et al., 2010), its accuracy is limited by the difficulty of outlining the cross‐section of a 3‐dimensional object on a 2‐dimensional video frame and by the fact that endoscopes have a degree of fisheye distortion, which means that pixels at the center of the image correspond to a different dimension than pixels in the outskirts of the image (Genta et al., 2016). Studies have shown a reasonable agreement between CSAs estimated from endoscopy and gold standard measurements (Calloway et al., 2013; Isono, Remmers, et al., 1997), but these validation studies are typically performed on a small number of geometries. There is a need to develop more accurate methods to estimate CSAs from endoscopy.

Third, the CPAP device used in this study did not have the ability to generate sub‐atmospheric pressures. In many cases, *P*_CLOSE_ was negative and thus had to be extrapolated rather observed directly, particularly after MAD advancement (Figure 7). While it is possible that the slope of the area–pressure curve may have been different outside the range of observations, for those cases where the range of observations included the closing pressure, the area–pressure relationship was linear in the entire range and the value of *P*_CLOSE_ estimated from the linear regression (Equation 2) was in good agreement with the observations.

Fourth, the pressure catheter used in this study had a single sensor at its tip, thus the intraluminal pressure could not be recorded at multiple sites. However, the operator adjusted the catheter position as needed to keep the sensor near the velopharyngeal site of collapse. Thus, our technique allowed us to quantify the area–pressure relationship at the velopharyngeal site of collapse.

Fifth, pressure oscillations associated with inspiration/expiration were not observed at some CPAP levels before MAD advancement in 3 of 16 patients despite a patent velopharynx. This was interpreted as complete airway obstruction distal to the velopharynx, so that intraluminal pressure at the velopharynx remained constant and equal to the nasal mask pressure. In these cases, the area–pressure measurements were performed at points where the noisy signal of the pressure catheter had a local minimum. This was observed only at lower CPAP pressures and there was no change in the slope of the tube law as compared to higher CPAP pressures where the pattern of inspiration/expiration was easily detected, thus we judged that the presence of distal obstructions did not affect our estimate of the velopharyngeal tube law.

Sixth, inspiratory airflow was not recorded in this study, thus *P*_CRIT_ was not quantified. *P*_CRIT_ and velopharyngeal *P*_CLOSE_ likely have similar values in patients with isolated collapse at the velopharynx, but these quantities will have different values in patients whose primary site of collapse is the epiglottis or oropharynx because in these patients airflow will fall to zero before the velopharynx closes. Since inspiratory airflow was not recorded in this study, we could not explore the relationship between *P*_CRIT_ and *P*_CLOSE_.

Seventh, the level of sedation was not recorded in our study. Patient 8 was an outlier with the inverse response (i.e., *P*_CLOSE_ increased after MAD advancement; Table 2). We speculate that this outlier behavior may be due to changes in the level of sedation. A deeper sedation after MAD advancement would predict lower muscle tone and higher *P*_CLOSE_. However, we could not test this hypothesis because the level of sedation was not recorded.

Finally, we found no correlation between P_CLOSE_ and AHI (Table 3). To our knowledge, the correlation between *P*_CLOSE_ and AHI was investigated by a single study to date, which also found no correlation between *P*_CLOSE_ and AHI, but found a significant correlation between the change in AHI and the change in *P*_CLOSE_ with MAD use (Ng et al., 2003). The lack of correlation between *P*_CLOSE_ and AHI in our study may be due in part to the fact that there are multiple sites of UA collapse in OSA patients, namely isolated velopharyngeal collapse, isolated oropharyngeal collapse, isolated epiglottic collapse, and simultaneous collapse of different structures. We measured *P*_CLOSE_ at the velopharynx only, whereas the site of collapse is variable among patients. Future studies are needed to test the hypothesis that velopharyngeal *P*_CLOSE_ correlates with AHI in a population of patients with isolated collapse at the velopharynx.

## 5 CONCLUSIONS

In summary, advancement of the mandible significantly reduced the velopharyngeal closing pressure and significantly increased the velopharyngeal CSA but did not have a statistically significant effect on velopharyngeal compliance. These results suggest that MADs reduce velopharyngeal collapsibility primarily by increasing airway size as opposed to affecting velopharyngeal compliance. This contradicts the speculation of previous literature that MADs stabilize the UA in part by stretching the soft palate and reducing velopharyngeal compliance. These findings suggest that quantification of velopharyngeal CSA pre‐ and post‐MAD advancement can be used as a biomarker to predict the success of MAD therapy. In our cohort, patients with hypopnea predominance had a lower *P*_CLOSE_ and patients with apnea predominance had higher *P*_CLOSE_, which suggests that patients with apnea predominance have a greater structural burden. Velopharyngeal compliance correlated with the fraction of respiratory events that were hypopneas, which previous literature suggests is a biomarker of arousability. Further research is needed to investigate this potential interplay between pharyngeal compliance and arousability.

## AUTHOR CONTRIBUTIONS

Guilherme Garcia: Study design, obtained funding, developed data acquisition system, patient recruitment, data collection, estimation of airspace cross‐sectional areas from endoscopic images, data analysis, statistical analysis, literature review, manuscript preparation, and revision of final manuscript. Josiah Wolf: Literature review, manuscript preparation, and revision of final manuscript. David Campbell: Estimation of airspace cross‐sectional areas from endoscopic images, and revision of final manuscript. Ryan Bailey and Charles Welzig: Developed data acquisition system and revision of final manuscript. Rodney Sparapani: Statistical analysis and revision of final manuscript. Tucker Woodson: Study design, obtained funding, patient recruitment, data collection, performed drug induced sedated endoscopy, literature review, manuscript preparation, and revision of final manuscript.

## CONFLICT OF INTEREST

The authors have no conflict of interest to disclose.
